# Tau Post-translational Modifications: Dynamic Transformers of Tau Function, Degradation, and Aggregation

**DOI:** 10.3389/fneur.2020.595532

**Published:** 2021-01-07

**Authors:** Carolina Alquezar, Shruti Arya, Aimee W. Kao

**Affiliations:** Department of Neurology, Memory and Aging Center, University of California, San Francisco, San Francisco, CA, United States

**Keywords:** phosphorylation, acetylation, ubiquitination, methylation, sumoylation, glycosylation, glycation, proteolysis

## Abstract

Post-translational modifications (PTMs) on tau have long been recognized as affecting protein function and contributing to neurodegeneration. The explosion of information on potential and observed PTMs on tau provides an opportunity to better understand these modifications in the context of tau homeostasis, which becomes perturbed with aging and disease. Prevailing views regard tau as a protein that undergoes abnormal phosphorylation prior to its accumulation into the toxic aggregates implicated in Alzheimer's disease (AD) and other tauopathies. However, the phosphorylation of tau may, in fact, represent part of the normal but interrupted function and catabolism of the protein. In addition to phosphorylation, tau undergoes another forms of post-translational modification including (but not limited to), acetylation, ubiquitination, glycation, glycosylation, SUMOylation, methylation, oxidation, and nitration. A holistic appreciation of how these PTMs regulate tau during health and are potentially hijacked in disease remains elusive. Recent studies have reinforced the idea that PTMs play a critical role in tau localization, protein-protein interactions, maintenance of levels, and modifying aggregate structure. These studies also provide tantalizing clues into the possibility that neurons actively choose how tau is post-translationally modified, in potentially competitive and combinatorial ways, to achieve broad, cellular programs commensurate with the distinctive environmental conditions found during development, aging, stress, and disease. Here, we review tau PTMs and describe what is currently known about their functional impacts. In addition, we classify these PTMs from the perspectives of protein localization, electrostatics, and stability, which all contribute to normal tau function and homeostasis. Finally, we assess the potential impact of tau PTMs on tau solubility and aggregation. Tau occupies an undoubtedly important position in the biology of neurodegenerative diseases. This review aims to provide an integrated perspective of how post-translational modifications actively, purposefully, and dynamically remodel tau function, clearance, and aggregation. In doing so, we hope to enable a more comprehensive understanding of tau PTMs that will positively impact future studies.

## Introduction

Post-translational modifications (PTMs) refer to the modifications that occur in a protein either shortly after its translation by ribosomes or after its folding and localization are complete (1). These modifications are usually catalyzed by enzymes and involve the addition of chemical groups, sugars, or proteins to specific residues of the targeted protein. Nearly all cellular processes can be regulated via PTMs. Therefore, PTMs provide a means for the natural increase in the proteome complexity and allow one gene to encode a variety of distinct protein molecules with distinct functional roles, known as proteoforms (2). PTMs alter the charge and hydrophobicity (electrostatics) of a protein, which in turn induces structural changes that influence protein function, protein-protein interactions, and protein aggregation (3, 4). In this way, PTMs also can affect the clearance of proteins by regulating the functionality of degrons, peptide sequences that target a protein for degradation (5). Essentially all proteins in eukaryotes, including the “intrinsically disordered” or “natively unfolded” proteins that tend to aggregate in neurodegenerative diseases, are susceptible to PTMs (3, 6, 7). Unlike folded proteins, natively unfolded proteins do not have a well-defined three-dimensional structure and exist as an ensemble of dynamically fluctuating conformations. The lack of tertiary structure makes natively unfolded proteins more vulnerable to PTMs (8).

**Figure 1:**
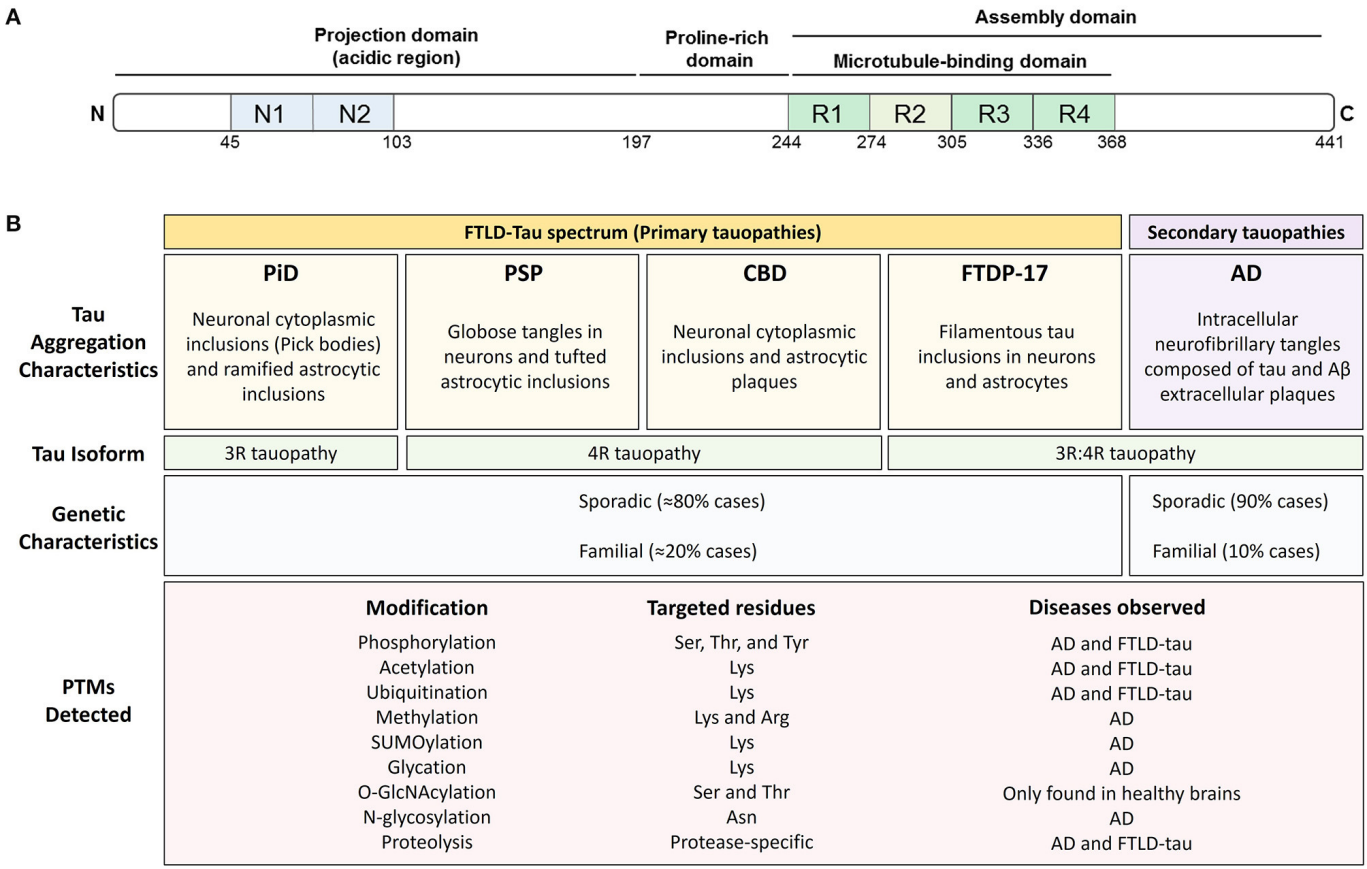
Post-translational modifications are found throughout tau and associate with all tauopathies. **(A)** Schematic representation of the structural features of 2N4R tau protein. Tau exists in 6 isoforms generated by the alternative splicing of exons 2, 3, and 10 of the MAPT gene. The isoforms include 0, 1, or 2 amino-terminal inserts (blue rectangles) and three (3R) or four (4R) microtubule-binding potential repeat domains (green rectangles). **(B)** Pathological and genetic classification of tauopathies as they relate to post-translational modifications (PTMs). Primary tauopathies fall under the umbrella pathological term frontotemporal lobar degeneration with tau inclusions (FTLD-Tau) and include Pick's disease (PiD), progressive supranuclear palsy (PSP), corticobasal degeneration (CBD), and frontotemporal dementia with parkinsonism (FTDP-17). Alzheimer's disease, a secondary or mixed tauopathy, is characterized by the presence of extracellular inclusions containing Amyloid β (Aβ) protein. Tauopathies are also classified depending on tau isoform composing the intracellular inclusions. PiD is a 3R-predominat tauopathy, PSP, and CBD are 4R tauopathies and FTDP-17 and AD are 3R:4R tauopathies. The PTMs observed in tau, the targeted residues and the disease where they have been observed is also shown.

Tau is a classic example of a natively unfolded protein (7, 9) that can be modified by a myriad of PTMs. Tau is a microtubule-binding protein found in neurons and glial cells, and is primarily involved in the stabilization of the cytoskeleton (10). Based on its interaction with microtubules and on the basis of its amino acid composition, the primary structure of tau can be divided into an N-terminal projection domain, a proline-rich region, a repeat region, and a C-terminal domain (11) (Figure 1). Tau has six different isoforms in human brain that differ from each other in the number of N-terminal inserts and either contain or lack one repeat (R2) of the four repeats (R1-R4) present in the microtubule-binding domain region. Because of its ability to bind to microtubules, the main function of tau is thought to be the modulation of microtubule dynamics, which consequently affects neurite outgrowth, axonal transport, and synapsis (12–14). Recent studies have shown that tau also interacts with several cell membranes, including the plasma membrane, the endoplasmic reticulum, and Golgi (15–17). Tau may also regulate intracellular signaling cascade via interactions with proteins such as 14.3.3, Pin-1, and Fyn (18), suggesting a role for tau in cell signaling (18). Although tau is primarily an axonal protein, it can be found within neuronal nuclei where it binds to either DNA or RNA and regulates gene expression (19–21). Under certain pathological circumstances, tau loses its function as a microtubule-binding protein and accumulates in the cytosol of affected cells, forming first “pre-tangles” and then insoluble cytosolic inclusions or aggregates composed of fibrillar forms of tau known as neurofibrillary tangles (NFTs).

Diseases associated with the presence of tau inclusions in the brain are collectively referred to as tauopathies and include a diverse group of neurodegenerative diseases with distinct pathological characteristics (Figure 1). Tau-immunopositive intracellular aggregates are the pathognomonic hallmark of tauopathies (22). However, the forces driving tau, especially non-mutant, wild-type tau, into intracellular aggregates are incompletely understood. The cellular protein homeostasis (or proteostasis) network is fundamental to the proper functioning of tau and prevents the accumulation of tau aggregates. In principle, proteostasis encompasses the entire life cycle of a protein such as tau, including synthesis, proper folding, post-translational processing, and degradation. Thus, PTMs are a key contributor to tau proteostasis and thereby could regulate tau function, levels, and aggregation.

The same PTMs that regulate tau function also have the ability to induce alterations in its clearance, conformation, and aggregation potential (23). However, not all tau PTMs are “pathological”. Many PTMs have been identified in tau extracted from healthy brains, suggesting a normal role for PTMs in tau function (24). Among all tau PTMs, phosphorylation is the most studied, and traditionally, it was thought that increased phosphorylation was the trigger for tau intracellular aggregation (25). However, a growing body of evidence suggests that other PTMs also considerably regulate tau function and may even precede phosphorylation in the sequence of events leading to tau inclusions formation.

In this review, we first provide a synopsis of the many tau PTMs that impact its function and potentially contribute to dysfunction (section Effects of PTMs on Tau Function and Dysfunction). We then fold this information into an integrated discussion of how PTMs can regulate tau degradation (section Effect of PTMs on Tau Degradation) and aggregation (section Effect of PTMs on Tau Solubility and Aggregation). A rarely discussed but critically important subject, cross-talk, and competition between PTMs, is considered next (section Cross-Talk and Competition Between PTMs). Finally, we summarize strengths and limitations of current approaches to the study of PTMs (section Approaches and Limitations) and provide concluding remarks (section Concluding Remarks).

## Effects of PTMs on Tau Function and Dysfunction

**Figure 2:**
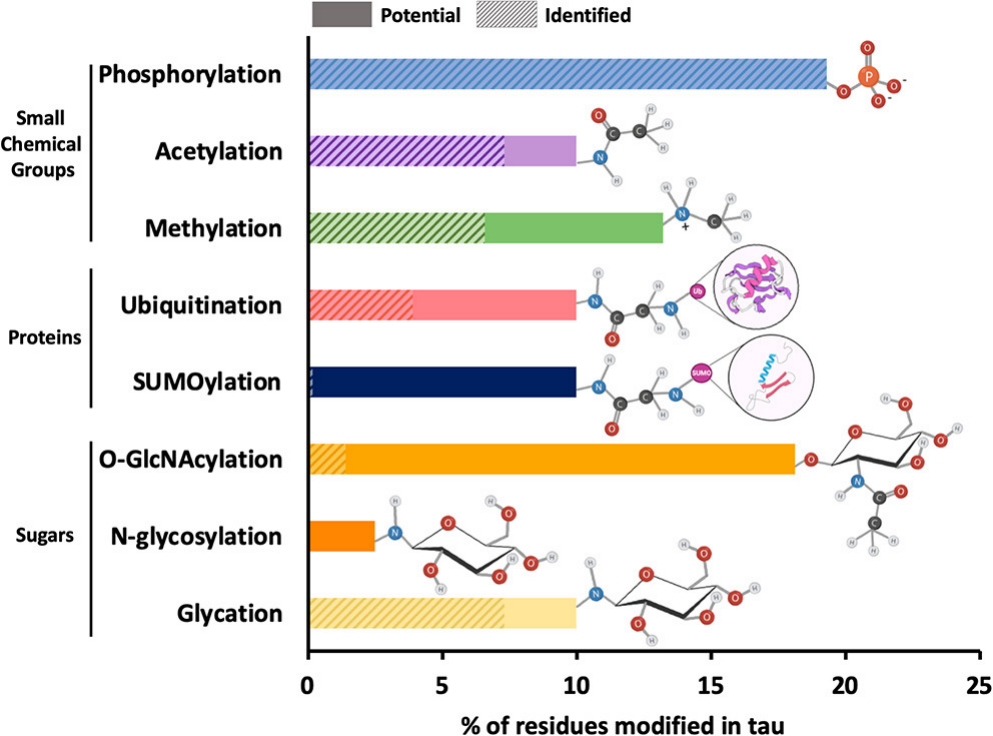
Relative frequency of post-translational modifications on tau. The 2N4R isoform of tau contains 441 amino acid residues, around 35 percent of which can potentially undergo PTMs. These residues include serine (forty-five), threonine (thirty-five), tyrosine (five), asparagine (three), arginine (fourteen), and lysine (forty-four). The most common PTMs in tau are shown on the y-axis of the bar plot. The x-axis of the plot shows the percentage of amino acid residues with solid bars showing the sites that have been identified within total potential sites shown in shaded bars. The functional groups associated with each PTM are also shown next to each bar. Since all potential phosphorylation sites have been identified, the shaded bar is completely masked by the solid bar. Similarly, in case of N-glycosylation, the specific N-glycosylation sites have not been reported and therefore, only potential sites represented by shaded bar have been shown.

The main role of PTM addition to any protein, including tau, is to regulate and increase functional diversity usually by altering electrostatics and/or structure. Approximately 35 percent of the amino acid residues in tau are susceptible to modification peri- or post-translationally. These residues are serine (S), threonine (T), tyrosine (Y), lysine (K), arginine (R), asparagine (N), histidine (H), and cysteine (C). Like other proteins, tau undergoes PTMs that involve the addition of small chemical groups or peptides on different side chains of tau: phosphorylation on S, T, or Y; acetylation, ubiquitination, SUMOylation, and glycation on K; methylation on K or R; O-GlcNAcylation on S and T, N-glycosylation on nitrogens, nitration on Y and oxidation on carbons (Figure 2). Tau can also undergo protonation on histidine residues and proteolytic cleavage (or truncation), although these modifications are often not considered alongside traditional PTMs. The implications of all of these modifications will be discussed in this section.

**Figure 3:**
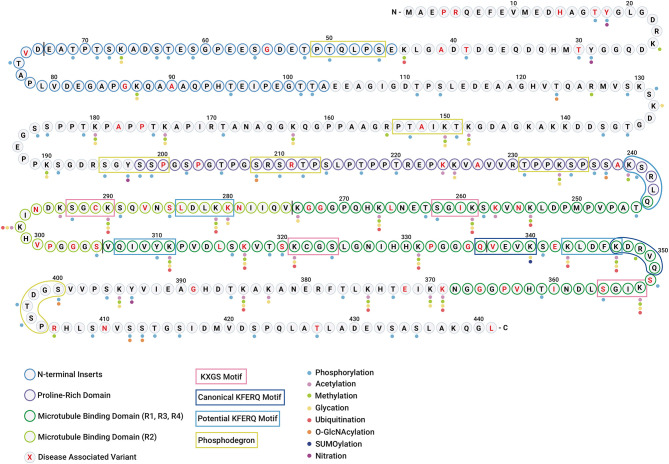
Tau undergoes diverse post-translational modifications that often compete for the same amino acid residues. Diagram showing the polypeptide chain of the longest tau isoform 2N4R tau along with individual amino acid residues highlighting different regions/domains. The N-terminal inserts are represented with blue residue borders, proline-rich domain is represented with purple residue borders, microtubule-binding domains are represented with green residue borders (R1, R3, R4 in dark green, and R2 in light green), and disease-associated amino acid variants are highlighted in red text. The vertical black line between residues is shown for distinction between two N-terminal inserts and four microtubule-binding repeats. The colored dots represent the PTMs identified in tau (WT and mutant) *in vitro* and *in vivo*. The color code for PTMs is as follows: phosphorylation (light blue), acetylation (pink), methylation (green), glycation (yellow), ubiquitination (red), O-GlcNAcylation (orange), SUMOylation (dark blue), and nitration (purple). N-glycosylation is omitted as no specific sites have been identified. The KXGS microtubule-binding motifs are shown in pink boxes. The canonical and potential KFERQ motifs are represented with dark blue and light blue boxes, respectively. The phosphodegron motifs are highlighted with green boxes.

The clustering of PTMs within short sequence motifs and functional domains suggests that tau function and subcellular localization could be regulated by a complex interplay between different PTMs (Figure 3). Since some modifications have been found only in the tau aggregates associated with disease (26, 27), it has been suggested that these modifications may be regulating tau dysfunction. Although a hypothetical sequence of events has been proposed to occur during the formation of tau inclusions (28, 29), what remains elusive is which PTMs actually drive tau self-association vs. those that are simply part of normal tau function and captured or trapped as “bystanders” in aggregates. The transformation from healthy (functional) to aggregated (or pathological) tau is most likely not a consequence of a single PTM, but rather a combination of the intrinsic structural alterations and extrinsic cellular conditions that may ultimately constitute pathogenicity. Below, we describe the PTMs that have been observed in tau and their potential impacts on tau function and dysfunction.

### Phosphorylation

Protein phosphorylation is one of the most common PTMs in the proteome (30). Phosphorylation involves the reversible addition of a phosphate (PO_4_) group to the polar group of serine, threonine or tyrosine amino acid residues (31) (Figure 2). Protein kinases and phosphatases, the enzymes responsible for phosphorylation and dephosphorylation, respectively (32), coordinate the phosphorylation status of proteins. Phosphorylation alters protein electrostatics by introducing a negatively charged, hydrophilic group, resulting in an overall more hydrophilic protein. As a consequence of this increased hydrophilicity, phosphorylation imparts conformational changes, and regulates some important protein functions including protein-protein interactions, signaling cascades, and protein degradation (5, 33). Phosphorylation is involved in essential cellular functions such as metabolism and intracellular signaling, and therefore, is fundamental to several normal cellular processes. However, abnormal phosphorylation has also been described as one of the primary causes for the alteration of a variety of structural, functional, and regulatory proteins under disease conditions.

The longest isoform of tau, known as 2N4R tau, has 85 potential phosphorylation sites (44 S, 35 T, and five Y) (34). Therefore, almost 20 percent of tau has the potential to become phosphorylated (Figures 2, 3). Of these sites, ~20 residues have been found to undergo phosphorylation in tau extracted from healthy brains (35, 36). In contrast, around 44 residues, some of which overlap with residues found in tau from healthy brains, have been identified as “abnormally” hyperphosphorylated in individuals with a tauopathy (25). Phosphorylation of serine and threonine residues is associated with the completion of degrons (or degradation-signaling motifs) and the subsequent clearance of tau (for a more extensive discussion about phosphodegrons, see section Effect of PTMs on Tau Degradation). If tau degradation is blocked, then these normally transient phospho-sites would persist, even though they are a part of the normal metabolism of tau. Thus, the phosphorylation observed in tau extracted from diseased brains could either occur abnormally as part of disease pathogenicity or be a found as a normal event to promote the normal, but interrupted, clearance of tau. Additionally, it is also possible that many other tau phosphorylation sites exist that have not been identified in post-mortem human brain material due to rapid dephosphorylation of tau (37).

As a dynamic and highly regulated process, tau phosphorylation requires a balanced interplay of kinases and phosphatases. Several serine/threonine kinases phosphorylate tau, including glycogen synthase kinase 3 (GSK3β), cyclin dependent kinase 5 (cdk5), casein kinase 1 (CK1), cyclic AMP-dependent protein kinase (PKA), p42/p44 MAPKs (ERKs 1/2), protein kinase C (PKC), calmodulin-dependent protein kinase II (CaMKII), the brain-specific kinases 1 and 2, the tau-tubulin kinases 1 and 2, and some microtubule affinity-regulating kinases (MARKs; also known as PAR1 kinases) (38). Furthermore, tau can be phosphorylated by tyrosine kinases, such as the SRC family members (LCK, SYK, and FYN) and the ABL family members (ARG and ABL1) (39). FYN, SYK, and ABL kinases phosphorylate tau at the Y18, Y197, and Y394 residues (40), which have been associated with tau aggregation and AD pathology (41).

Tau can be dephosphorylated by protein phosphatases (PP) 1, 2A, 2B, 2C, and 5 (25). Among these phosphatases, PP2A accounts for around 70 percent of all tau dephosphorylation in the human brain (42). Interestingly, PP2A also dephosphorylates and modulates the activity of components along the ERK1/2 MAPK cascade that leads to GSK3β activation (43), suggesting potential feedback cycles. As phosphorylation is one of the most studied PTMs in tau, the kinase pathways implicated in tau phosphorylation have been extensively reviewed elsewhere (44). The regulation of the phosphorylation status of tau is essential to maintain the biological function of the protein.

The change in tau electrostatics associated with phosphorylation may have a critical role in the regulation of tau function, localization, and interaction with other molecules. The phosphorylation status of tau is developmentally regulated, implying the significance of phosphorylation in the regulation of specific function of tau in each developmental stage (45, 46). Tau phosphorylation inside the microtubule-binding domain (e.g., S262/S214) and/or the proline-rich (e.g., T231) domain reduces the affinity of tau for negatively charged microtubules (47–50). Binding of tau stabilizes microtubules, therefore tau phosphorylation regulates neuronal functions such as axonogenesis and neurite outgrowth (47, 51–53). Additionally, it has been reported that the specific phosphorylation of tau by FYN kinases induces the relocation of tau from the axon to dendrites (54). However, the effects of tau relocation in neuronal function are not yet well-understood. Additionally, tau interacts with the kinesin-associated protein JIP1 (JUN N-terminal kinase-interacting protein 1) only when phosphorylated, thereby impairing the formation of the kinesin complex that mediates axonal transport (55).

Since phosphorylation regulates the biological functions of tau, inappropriate phosphorylation may be an important contributor to the pathogenesis of tauopathies. The abnormal phosphorylation in several residues inside the KXGS motif (25) and the proline-rich domain (47, 56–58) has been identified in NFTs of tauopathy patients brains. Aberrant tau phosphorylation at these motifs may pose detrimental consequences for neurons, such as the impairment of neurite outgrowth (59) and the deregulation of axonal transport (60, 61), which may lead to synaptic dysfunction and neurodegeneration (62–64). The phosphorylation at residues outside of the microtubules-binding and proline-rich domains does not interfere with the ability of tau to bind microtubules. Some of these residues such as S396, S404, and S422, have been found abnormally “hyperphosphorylated” in diseased brains (57, 65), and it has been suggested that phosphorylation at these residues may influence tau aggregation (66). While substantial published work suggests that tau phosphorylation may contribute to development of tauopathies, the exact mechanism behind how phosphorylation regulates tau function and aggregation *in vivo* is still unknown. To unravel the role of abnormal tau phosphorylation in tauopathies, it will be necessary to better understand the broader impacts of phosphorylation on normal tau function.

### Acetylation

The acetylation of mammalian proteins involves the N-terminal addition of an acetyl group from acetyl coenzyme A (Ac-CoA) to the lysine residue of a polypeptide chain (67) (Figure 2). Acetylation was first described on histone proteins, therefore the associated enzymes are known as histone acetyl transferases (HATs) and histone deacetylases (HDACs), although they are less discriminating in their targets than their names would suggest (67). Acetylation is considered an essential modification that plays critical roles in the function of diverse proteins, including p53 and tubulin (68). The addition of an acetyl group neutralizes the positive charge of lysine residues, thereby eliciting its downstream molecular impacts on protein structure and protein-protein recognition. Among its functional roles, acetylation is perhaps best known to regulate gene expression via modification of histone structure and chromatin accessibility (69). However, the acetylation of non-histone proteins regulates a range of cellular processes such as DNA damage repair, cell division, signal transduction, protein folding, autophagy, and metabolism (70). Incorporation of an acetyl group onto lysine residues prevents other modifications and thus, acetylation also can block the processes regulated by ubiquitination, SUMOylation, methylation, and glycation.

Acetylation has recently arisen to challenge the predominance of phosphorylation as the key PTM on tau. The longest isoform of tau protein (2N4R) contains 44 lysine residues and thus, 10 percent of the protein has the potential to be modified by acetylation (Figure 3). Among all HATs implicated in protein acetylation, p300 and its close homolog CREB-binding protein (CBP) appear competent to acetylate tau *in vitro* (71, 72). Moreover, the histone deacetylase 6 (HDAC6) (36, 73) and the NAD^+^-dependent sirtuin 1 deacetylase (SIRT1) (72) are the major tau deacetylases. Interestingly, these two HDACs have seemingly opposing effects on tau: while tau deacetylation by SIRT1 has a protective role against tau accumulation, the deacetylation of tau by HDAC6 contributes to the increase of tau phosphorylation and aggregation *in vitro* (74, 75). Tau has also been shown to undergo acetyl-coenzyme A–induced auto-acetylation *in vitro* (76). However, the role of tau as acetyltransferase remains somewhat controversial, given that other groups have not consistently identified tau acetylation in absence of HAT enzymes (26, 76, 77).

Tau acetylation was first recognized in the context of neurodegeneration in mouse models of neurodegenerative disease (72, 78), and subsequently in brain lysates from AD patients at early/moderate Braak stages (79). The dysregulation of the HATs and HDACs involved in tau acetylation was also found in diseased brains (79, 80). However, the underlying abnormalities of HAT and HDAC activity may be both context and disease specific. In this regard, while increased p300/CBP HAT activity was observed in the brains of FTLD-tau patients (80), p300/CBP levels, and activity were found to be lower in the frontal cortex and hippocampus of AD patients (81) and in a mouse model of AD (82). Although these reports failed to show the effect of p300/CBP dysregulation in tau acetylation, they suggest that the role of p300/CBP in tau acetylation may vary between different tauopathies such as FTLD-Tau and AD. Furthermore, the levels and activity of HDACs have also been measured in patients' brains. It has been observed that the expression of SIRT1 is reduced in AD patients (83, 84) while the levels of HDAC6 are increased (73), confirming that these enzymes have opposite effects on tau function and dysfunction. Altogether, this evidence demonstrates that tau acetylation may have a key role in tauopathies.

As with phosphorylation, to fully understand the role of acetylation in tau dysfunction, one must necessarily understand its role in the normal function of tau. While the neutralization of positively charged lysine residues by acetylation appears crucial for regulation of tau, the downstream functional impacts of this regulation seem to be residue specific. For example, acetylation at lysine residue K280 weakens the binding of tau to negatively charged microtubules, potentially destabilizing microtubule networks (26, 27, 85). This contrasts with acetylation at K174, which induces tau aggregation without affecting tau-microtubule binding (79). Finally, HDAC6-regulated tau acetylation at KXGS motif-associated K259, K290, K321, or K353 residues suppresses both tau phosphorylation and aggregation (36). Unlike acetylation of lysine residues that have been identified only in tauopathy brains (26, 79, 86, 87), the acetylation at these four residues occurs in normal tau but is reduced in tauopathy brains (36). Since acetylation has emerged as a key PTM in the regulation of tau function and dysfunction, mapping all the acetylation sites on tau in different biological contexts will help to delineate the ultimate roles and consequences of each modification.

Acetylation on tau is particularly interesting because many lysine residues that are targets for acetylation are also targets for other PTMs, including ubiquitination, SUMOylation, methylation, and glycation (78, 88, 89) (Figure 3). Therefore, a potential rivalry between PTMs could exist, where the addition of one chemical group to a given residue blocks the addition of another, suggesting multilayered avenues for the regulation of the biology of the protein (88). While the cross-talk between PTMs will be extensively discussed in section Cross-Talk and Competition Between PTMs, below we review the role of other lysine-associated PTMs in tau function.

### Ubiquitination

Covalent modification of proteins with ubiquitin (Ub) (ubiquitination or ubiquitylation) represents one of the most common PTMs in mammalian cells (Figure 2). The small, 76 residues, protein ubiquitin is nearly ubiquitously found in eukaryotes (ergo its name) and is used to modify activity, localization, or stability of other proteins (90, 91). Ubiquitination involves a multi-step process carried out sequentially by activating (E1), conjugating (E2), and ligating (E3) enzymes and has been reviewed elsewhere (92). Similar to acetylation, ubiquitination occurs on lysine residues. In case of ubiquitination, the C-terminal carboxyl group of a lysine in the ubiquitin sequence itself is covalently attached to the ε-amino group on a lysine of the “target” protein. Substrates can be modified with a single ubiquitin (monoubiquitination) or with polymeric Ub chains of variable length (polyubiquitination). The ubiquitin protein contains seven lysine residues. Therefore, the nomenclature around ubiquitination is based upon which of these seven lysine residues serves as the initial link to the target protein (i.e., ubiquitinated chains are called K6, K11, K27, K29, K33, K48, or K63) (93). The type of ubiquitin chain determines the biological effects of these modifications. Chains linked through K6, K27, and K33 are often involved in cell proliferation, DNA damage repair, and innate immunity (91). Other linkages are directly related with protein degradation. In this regard, K48 chains generally target substrates for degradation by the 26S proteasome (94), whereas K63-polyubiquitinated proteins are directed to the degradation by the autophagy-lysosomes pathway (93–96).

As a protein rich in lysine residues, tau has a high susceptibility toward ubiquitination. Out of a total of 44 lysine residues, 17 residues of the 2N4R tau isoform have been found ubiquitinated with most of them located in the tau microtubule-binding domain (78, 97). The main role of tau ubiquitination appears to be the regulation of tau clearance by the proteasomal or lysosome-autophagy systems (98). E3 ligases confer ubiquitin residues to target proteins (99). Among ~600 E3 ligases encoded by the human genome, only three appear competent to ubiquitinate tau: the C-terminus of the Hsc70-interacting protein (CHIP), the TNF receptor-associated factor 6 (TRAF6), and axotrophin/MARCH7 (100–102). Each of these E3 ligases ubiquitinates tau through different linkages and at different residues, suggesting that each ligase modulates tau degradation by different mechanisms. CHIP ubiquitinates tau through K48 or K63 linkages and thus, regulates tau degradation via both proteasomal and autophagy systems (102–104). On the other hand, the E3 ligase TRAF6 ubiquitinates tau via K63 linkages (100), suggesting that ubiquitination mediated by this enzyme may regulate the degradation of tau in the autophagy-lysosomes pathway only (100, 105–107). Additionally, *in vitro* experiments have shown that tau can be monoubiquitinated by axotrophin/MARCH7 (101). However, the effect of monoubiqutination by axotrophin/MARCH7 on tau function and degradation is not yet well-understood.

While E3 ligases attach ubiquitins, deubiquitinases (dUbs) remove them. The cysteine protease Otub1 is the only dUb that has been shown to target tau. Outb1 removes the K48 polyubiquitin chains from endogenous tau, preventing tau degradation in primary neurons derived from a transgenic mouse model (108) and thus implying an important role in the regulation of tau ubiquitination.

Ubiquitin has been found in aggregated tau extracted from the brains of tauopathy patients. However, whether ubiquitin is causal, contributory, or simply a bystander in aggregation remains to be seen. Tau isolated from human AD brains has been shown to be monoubiquitinated at K254, K257, K311, and K317 (109) and polyubiquitinated at K254, K311, and K353 residues (110). Tau was also found to be ubiquitinated at K290 in a mouse model of AD (78). Interestingly, insoluble tau from AD brains is modified predominantly by K48 linkage (110), whereas soluble tau can also be ubiquitinated via K63 polyubiquitin conjugation (102), suggesting that soluble and aggregated tau are degraded by different pathways. While ubiquitin is a component of tau aggregates found in the brains AD patients (109, 111–113), tau “pre-tangles” did not exhibit positive ubiquitin immunostaining (114–116). Moreover, it has been shown that tau phosphorylation precedes ubiquitination in the NFTs of AD patients and that the formation of paired helical filaments precedes tau ubiquitination *in vitro* (109, 114), suggesting that ubiquitin may be linked to tau after the formation of the fibrillar inclusions. This finding potentially supports ubiquitination as a compensatory response to tau accumulation. In contrast, other groups have reported that both mono- and polyubiquitination contribute to the formation of insoluble protein inclusions present in neurodegenerative diseases (96, 117) and that the induction of tau ubiquitination in cell cultures increases aggregation (102). The somewhat contradictory nature of the current literature around ubiquitination suggests that whether this modification is causal, consequential, bystander, or context specific in disease remains to be sorted.

### SUMOylation

In SUMOylation, the small ubiquitin-like modifier protein SUMO is transferred to the terminal amino group of lysine side chains of the target protein by an ATP-dependent enzymatic cascade. Analogous to ubiquitination, this SUMOylation cascade involves an E1 activating enzyme, an E2 conjugating enzyme, and an E3-type ligase (Figure 2) (118, 119). SUMO groups can be removed by specific proteases known as SUMO-specific proteases/isopeptidases (SENPs). Three main SUMO isoforms are expressed in cells: SUMO1, SUMO2, and SUMO3, of which SUMO2 and SUMO3 are more similar to each other and are different from SUMO1 (120).

SUMO is predominantly found in the nucleus and thus, SUMOylation plays a crucial role in many nuclear processes such as gene expression, genome stability (121), DNA damage response (122), protein trafficking (123), and cell cycle control (124). SUMOylation has emerged as an essential regulator of neuronal function with a growing evidence suggesting that SUMOylation of proteins inside and outside the nucleus plays an important role in neurodegenerative diseases (125). Alterations in protein SUMOylation are observed in a wide range of neurological and neurodegenerative diseases including tauopathies (126–128), and several extranuclear disease-associated proteins including tau have been shown to be directly SUMOylated (125).

*In vitro* studies have demonstrated that tau can be SUMOylated. Specifically, it has been found that tau is monoSUMOylated *in vitro* at the K340 residue, which is located within the microtubule-binding domain (128, 129). Tau becomes available for SUMOylation only after it is released from microtubules (129), suggesting that SUMOylation may be a secondary PTM that occurs after phosphorylation and/or acetylation. However, little is known about how SUMOylation interferes with the formation of tau inclusions in tauopathies. Tau SUMOylation at K340 inhibits tau ubiquitination and the subsequent proteasome-dependent degradation, suggesting that SUMOylation may block tau degradation and therefore induces tau accumulation and aggregation (128). In contrast, previous evidence indicates that the inhibition of the proteasome pathway stimulates tau ubiquitination and eliminates tau SUMOylation (129), suggesting that SUMOylated tau can be degraded by other pathways such as autophagy. In this context, a more recent study has demonstrated that SUMO1 labels lysosomes in oligodendrocytes from PSP patients containing tau inclusions (127), an indicator that SUMOylation may be regulating autophagy-lysosomes pathway in tauopathies. Additionally, the activation of autophagy-lysosomes pathway appears to reduce tau SUMOylation, tau inclusions and cortical atrophy associated with the rTg4510 mouse model of tauopathy (130). Despite the role of SUMOylation in tau function and dysfunction not being completely understood, substantive evidence support SUMOylation competing with ubiquitin for lysine residues in tau, with consequences on tau clearance in the proteasome and/or autophagy-lysosomes systems.

### Methylation

Methylation is a biochemical process that involves the transfer a methyl group (CH_3_) to DNA or protein targets (Figure 2). DNA methylation was discovered contemporaneously with the discovery of DNA as genetic material (131, 132). On DNA, methylation is regarded as an epigenetic means of regulating gene expression through blocking transcription factor binding or recruiting transcriptional repressors (133). Protein methylation was first described in histones. This was followed by decades of limited interest until the physiological role for protein methylation was documented in the late 1990s and non-histone proteins were also found to be methylated (134). Currently, methylation is fourth on the list of PTMs in terms of overall abundance in the proteome, with more than 18,000 methylation sites described in ~7,400 proteins (135). The N-methylation of histone and non-histone proteins occurs primarily on lysine and arginine residues. In the process of DNA or protein methylation, methyl groups from an S-adenosyl methionine (SAM) donor are conferred by methyltransferases and removed by demethylases (136). Lysine residues can be methylated up to three times, resulting in a mono-, di-, or trimethyl-lysine, which each give rise to distinctive biological consequences. Indeed, although the methyl group is one of the smallest post-translational modifications, each methylation event removes a proton from the ε-amino group and thereby decreases the hydrogen-bonding potential of lysine. Therefore, methylation also increases the hydrophobicity and bulkiness of lysine side-chains. Based on the electrostatic properties of methylation, this PTM has been shown to regulate different aspects of proteins function such as protein–protein and protein–nucleic acid interactions, protein stability, subcellular localization, and enzyme activity, which in turn affect essential cellular processes including transcription, protein synthesis, signal transduction, and metabolism (137, 138).

Tau methylation is a relatively recent discovery (89). Results from mass spectrometric analyses show that several lysine residues are methylated on tau extracted from either healthy individuals or AD patient brains (89, 139–141). Moreover, in mouse models tau is also methylated at arginine residues R126, R155, and R349 (78). The number of methyl residues in tau protein is relatively low compared to other modifications such as phosphorylation and acetylation (140). Tau from both healthy and AD patient brains can be mono and dimethylated (141, 142). Interestingly the methylation status of tau changes qualitatively with aging and disease progression (89, 140, 143). Inherent in this finding is the possibility that aging alone, rather than disease *per se*, dictates tau methylation, analogous to the “Aging Clock” described for DNA methylation (144).

Until now, the specific enzymes involved in tau methylation have not been identified, and the exact role of methylation in tau function and pathogenesis is unclear. Methylation changes the electrostatics of tau protein. Given that tau is methylated within the KXGS motifs, regions that are essential for interactions with microtubules, lysine methylation may suppress tau's microtubule-binding function (139). Since tau aggregates from AD patients are methylated (89), it has also been suggested that methyl residues can bind to tau after aggregation and perhaps impair ubiquitination and UPS-mediated degradation (89). Future investigation of site-dependent tau methylation *in vivo*, and determination of its relationship to the DNA methylation “Aging Clock”, would advance our understanding of the role of this modification in tau function and pathology.

### Glycosylation and Glycation

Glycosylation involves the addition of carbohydrate chains to proteins and lipids, of which we will focus on the former. Protein glycosylation is classified into two subtypes depending on the type of reaction involved: enzymatic glycosylation and non-enzymatic glycosylation (aka glycation). Enzymatic glycosylation typically occurs on secreted proteins or those that remain in membrane-bound organelles, hence this process occurs in the endoplasmatic reticulum (ER)/Golgi system. In addition, a specialized enzymatic glycosylation called O-GlcNAcylation occurs almost exclusively on cytoplasmic and nuclear (non-secreted) proteins (145). Given that the addition of sugar groups has steric effects on proteins, both enzymatic and non-enzymatic glycation have been described to play a major role in determining the structure and stability of proteins (146). However, while enzymatic glycosylation is an important PTM that exerts functional effects on glycoproteins, glycation typically results in dysfunctional or defective biomolecules (146, 147). Both enzymatic and non-enzymatic glycosylation of tau function are discussed below.

#### Enzymatic Glycosylation

Enzymatic glycosylation is a process in which glycosyltransferase enzymes attach activated sugar donor groups (monosaccharides) to proteins via covalent, glycosidic linkages (148–150). Sugar groups are linked to either asparagine (N-glycosylation) or serine/threonine (O-glycosylation) residues (Figure 2) (146). N-glycosylation begins as a co-translational event in the ER. There, the oligosaccharyltransferase enzyme adds an immature polysaccharide to a nascent polypeptide chain. After the further maturation of this polysaccharide, the resulting glycoprotein is transferred to the Golgi apparatus, where the sugar groups acquire an even more complex structure during a process called “terminal glycosylation” (149). Conversely, O-glycosylation is achieved post-translationally in the cis-Golgi compartment (151). N- and O-glycosylation also differ in the linker-residue of the targeted protein. N-glycosylation is characterized by the enzymatic addition of N-glycans to asparagine residues in the sequence N-X-S/T (where X is any amino acid except P or D), being X any amino acid except proline (149). O-glycosylation occurs on S/T residues with a β-turn in the proximity of a proline residue, however the O-glycosylation domains are not exactly defined (152). Both N- and O-glycosylation impact the structure, stability, folding, oligomerization, and solubility of glycoproteins (143, 153, 154).

Some proteins can additionally undergo a specialized and highly dynamic form of O-glycosylation called O-GlcNAcylation or *O*-GlcNAc. O-GlcNAcylation differs from other types of glycosylation in that the glycan is not further processed after the addition of a single GlcNAc to S/T residues. It also occurs almost exclusively on intracellular (nuclear and cytoplasmic) rather than secreted proteins (151). O-GlcNAcylation is highly regulated by two enzymes, O-GlcNAc transferase (OGT) and O-GlcNAcase (OGA) that add and remove sugar groups, respectively (145). Because O-GlcNAcylation affects S/T residues, many known O-GlcNAcylation sites are nearby or overlapping with phosphorylation sites and thus it has been suggested that these two post-translational modifications can regulate each other (145, 155–159).

Enzymatic glycosylation may contribute to tauopathies by modifying proteins other than tau, such as APP and the β-site APP-cleaving enzyme 1 (BACE1). APP and BACE1 have been found to be O-glycosylated (160–162) and N-glycosylated (163, 164) in tauopathy patients' brains. Tau also has the potential to be modified by N-glycosylation and by the specialized form of O-glycosylation, O-GlcNAcylation (165, 166). Interestingly, to date tau has been found to be N-glycosylated only in AD subjects (167–169), while O-GlcNAcylation levels are relatively elevated in brains from normal controls (155, 166, 170). To date, at least five O-GlcNAc sites have been mapped in tau (T123, S208, S238, S400, and one of S409/412/413) (171–173) and all of them are also susceptible to phosphorylation. Therefore, it has been speculated that O-glycosylation may compete with or protect from phosphorylation (155, 174). Since tau O-GlcNAcylation is decreased in diseased brain (155, 170), it has been suggested that this PTMs has a protective role. This hypothesis is potentially supported by the fact that that O-GlcNAcylation increases the interaction of tau with microtubules (175), increases tau degradation (166), and suppresses tau aggregation (171, 175, 176).

Despite tau being N-glycosylated, the exact residues have not been identified. Furthermore, the relevance of N-glycosylation in tau function is a matter of debate since tau is a cytosolic protein, but the N-glycosylation process occurs in the ER-Golgi system (177). Therefore, tau N-glycosylation is thought to be associated with aberrant subcellular localization of tau. However, the mechanisms and consequences behind tau relocation are still elusive. It has been proposed that N-glycosylation precedes and accelerates tau phosphorylation (169), either by suppressing tau dephosphorylation (178) and/or inducing tau phosphorylation by protein kinase A (PKA) (169, 178). While a direct impact of tau N-glycosylation on microtubule polymerization and stability has not been observed (167), the removal of N-glycans and phosphate groups of tau seems to restore its microtubule polymerization activity (164). Although little is known about the role of N-glycosylation in tau biology, this evidence suggests that aberrant N-glycosylation of tau may be involved in neurodegenerative disease pathogenesis.

#### Glycation or Non-enzymatic Glycosylation

Glycation (or non-enzymatic glycosylation) is a PTM in which sugars or sugar-derived metabolites are covalently attached to the side chain of lysine residues (Figure 2). The glycation process involves a set of chemically heterogeneous modifications that lead to the formation of advanced glycation end-products (AGEs) (179). AGEs are produced by the irreversible cross-link between glycated and non-glycated proteins and are considered glycotoxins with significance in aging and age-related diseases (180–182). Both early stage glycation and AGEs have been shown to impair protein activity. Moreover, AGEs deposition has been shown to correlate with age and disease progression in AD (183). Although the study of AGEs is an active area of investigation in the field of aging research (184), it is still unknown how proteins are selected to be glycated and the biological role of glycation in neurodegeneration is not well-understood (185).

Thirty-two lysine residues in 2N4R tau have been identified *in vitro* as potential glycation sites (186, 187) (Figure 3). Although most of these sites were detected in both 3R and 4R isoforms, K280 and K281 are absent in the 3R tau, and this difference seems sufficient to slow the glycation of 3R relative to 4R isoforms (186, 187). In AD, glycation has been detected in aggregated tau purified from human AD brains but not in soluble tau (188). The molecular functions for glycation, the sites of these reactions and whether or how they contribute to tau aggregation remain subjects for debate. Since the glycation of tau in microtubule binding domains reduces tau affinity for microtubules *in vitro* (189), it has been suggested that glycation sterically blocks tau-microtubule interactions. Glycation may also induce the accumulation of tau by blocking the ubiquitination of tau at lysine residues and consequently its degradation (189, 190). Furthermore, the late glycosylation product known as AGEs have also been found colocalized with pathological tau aggregates in the brain of sporadic AD cases (191). Similarly, AGEs colocalize with NFTs from PSP, PiD, and ALS patients (180, 191). Although, the role of glycosylation in tau function is not well-understood, evidence presented here implies that this PTM can play an important role in tauopathies.

### Proteolysis

Proteolysis refers to the breakdown of proteins into peptides and amino acids through the hydrolysis of peptide bonds via the action of proteases. Most proteases are highly specific and cleave their protein substrates from the N-terminus (aminopeptidases), C-terminus (carboxypeptidases), or more centralized regions (endopeptidases) of a protein. Furthermore, based on their catalytic mechanisms for substrate hydrolysis and the residues associated with their active sites, proteases are categorized into six types: aspartyl, cysteine, glutamic, metallo, serine, and threonine proteases. Proteolysis is a critical modification that can lead to alterations in protein function with important outcomes in many biological processes including signaling pathways and apoptosis. It can regulate the concentration of a protein, transform a protein into an active form, or process a protein to provide the amino acids required to synthesize a different protein. Proteolysis also plays an important function in removing damaged or unnecessary proteins from cells.

Several cytosolic proteases responsible for tau proteolysis have been identified, including caspases, calpains, and thrombin (192). Caspases are a family of evolutionarily conserved cysteine-dependent proteases that cleave proteins after specific aspartic acid residues (193). These proteases play a crucial role in important biological process such as apoptosis and inflammation (194). Thirteen different caspases have been described in humans, and their functional classification is reviewed elsewhere (193).

The role of proteolysis in tau function is incompletely understood. Tau proteolysis could be protective and may promote the removal of abnormal tau or could favor the abnormal accumulation of cleaved tau in the cell (195). It is also possible that tau cleavage is neither protective nor damaging, but is merely a bystander effect of tau accumulation. The proteolytic cleavage of tau has been observed both *in vitro* and *in vivo* (192, 195). Prior research on tau proteolysis has also shown that the proteolytic cleavage of tau precedes its aggregation *in vitro* (196–198). Furthermore, the N-terminal truncation of tau has been shown to alter the cellular localization of the protein from the cytosol to the nucleus (199), indicating a possible link between proteolysis and tau localization. Given that it has been shown that tau fragments induce death in cultured neurons (200), it has been suggested that tau cleavage may induce neurodegeneration. However, the toxic nature of tau fragments has not been demonstrated *in vivo* and therefore, it remains unclear if these fragments are indeed toxic.

The *in vitro* cleavage of tau by caspase-3,−7,−8, and less efficiently by caspases-1, and−6 resulting in the formation of an N-terminal fragment, tau1-421 (Tau-C3) (198, 201, 202) has been demonstrated. Tau-C3 fragment has been observed in AD brains (198) and interestingly, the active forms of both caspase-3 and caspase-6 are elevated in AD brains compared to control brains (203–205). While the exact function of Tau-C3 fragment is unknown, it has been shown that Tau-C3 fragment induce toxicity in neuronal cultures (201, 206, 207), and that the caspase-mediated tau cleavage precedes tangle formation in tau transgenic mice (Tg4510) (208). However, whether caspase-mediated cleavage of tau is toxic or protective for the neurons still remains a matter of debate.

While the proteolytic cleavage of tau by caspases has been extensively studied, the cleavage of tau by calpains and thrombin remains elusive. Calpains are calcium-activated cytosolic cysteine proteases implicated in a variety of calcium associated cellular functions like cell proliferation, migration, invasion, apoptosis, and signal transduction (209). Dysregulation of calcium homeostasis has been proposed to induce abnormal activation of calpains in a number of neurodegenerative diseases (209). The cleavage of tau by calpains, has been demonstrated *in vitro* and in neuronal cultures (210, 211). According to a recent study, calpain-mediated cleavage events are considered a part of normal tau protein processing (212). However, there are studies that have suggested that calpain-mediated proteolysis of tau may induce toxicity, as seen in cell cultures and a *Drosophila* tauopathy model (210, 213). Thrombin is a cytosolic serine protease that is expressed within neurons and astrocytes (195). The cleavage of tau by thrombin has been shown *in vitro* and in cell models of tauopathy (214, 215). Based on cell culture-based studies, thrombin cleavage products of tau are believed to be potentially pathogenic (216, 217). However, due to contradictory reports and lack of *in vivo* evidence for tau proteolysis mediated by these enzymes, the relevance of thrombin, and calpains in tau function remains unclear. Naturally, tau cleavage also occurs in the proteasome and lysosomes, which will be discussed further in section Effect of PTMs on Tau Degradation.

### Other PTMs

In addition to the PTMs discussed above, several other post-translational modifications occur on tau, which are less well-recognized. Below, we discuss three different tau PTMs that fall in this category: protonation, oxidation, and nitration.

#### Protonation

Protonation involves enzyme-independent, rapid, and reversible addition or removal of protons. Protonation adds a positive charge to otherwise uncharged amino acid side chains and therefore can lead to dynamic changes in protein conformation and function (218). The optimum protonation state of a protein is critical to its functioning. Dysregulation in local pH can lead to disruption in the protonation state of a protein and has recently been shown to have implications with neurodegenerative diseases (219, 220). Among several protein sites that can participate in the protonation/deprotonation process, only a few can significantly undergo this modification within the normal cytosolic pH range. Histidine side chains, with a nominal pKa of ~6.5, are among the most attractive residues for protonation within cytosolic pH range.

Tau contains highly-conserved histidine residues near the C-terminus of each microtubule-binding tau repeat (Figure 3) (221). These residues have been proposed to act as pH sensors and influence tau–microtubule interaction within the physiological intracellular pH range (221). The histidines are positively charged at pH <g7.5 and show increased tau-microtubule binding (221). However, at pH >7.5, histidine residues in tau are deprotonated and bind microtubules with lower affinity (221). Furthermore, it has been proposed that a highly conserved histidine residue, H299 (Figure 3), near the R2 C-terminus seems to contribute to tau–microtubule binding. The labile nature and minimal molecular weight change of protonation renders it difficult to detect by traditional approaches, such as mass spectroscopy or gel electrophoresis, used to detect PTMs. Nonetheless, given its effects on protein structure, solubility and function, protonation should be considered as a post-translational protein modification like the others in this review.

#### Oxidation

Like protonation, protein conformation is also sensitive to oxidation-reduction (redox) changes. Redox homeostasis is achieved by the regulation of appropriate levels of reactive oxygen (ROS) and nitrogen (RNS) species, that are considered important physiological regulators of intracellular signaling pathways (222). The dysregulation of ROS is a major contributor to oxidative damage and it has been proposed that age-related accumulation of oxidized proteins may contribute to the aging process (223). However, the importance of protein oxidation in the progression of aging still remains poorly understood. Cysteine residues are the prime amino acid that can exist in oxidized or reduced states. The unique properties of cysteine side chain allows it to undergo various oxidative PTMs, which can potentially have diverse regulatory effects. The oxidation of cysteine residues leading to disulfide bond formation is one of the well-established mechanisms underlying the redox regulation of protein conformation and hence its function.

On tau, there exists a pair of cysteine residues that can undergo oxidation (Figure 3). While the effect of oxidation on tau function is unknown, its role in tau aggregation has been studied *in vitro* (224) and is discussed in section Effect of PTMs on Tau Solubility and Aggregation of this review. Tau oxidation has only been described *in vitro*, and therefore the status of oxidized tau *in vivo* remains unclear.

#### Nitration

Nitration involves the addition of nitrogen dioxide (NO_2_) onto tyrosine residues. The mechanism underlying protein nitration is not clear but it most likely involves the presence of ROS/RNS-like peroxynitrite and therefore nitration can also occur during oxidative damage (225). Several lines of evidence suggest a role of nitration in physiological processes like signal transduction (226, 227). Additionally, increased protein nitration has been identified in a large variety of diseases and have also been implicated in the process of aging (227).

The 2N4R tau isoform has five tyrosine residues, and the nitration at four of them has been shown *in vitro*: Y-18, Y-29, Y-197, and Y-394 (Figure 3). These studies have shown that residues Y-18 and T-29 are more susceptible toward nitration than residues Y-197 and Y-394. The nitration of tau at Y-197 has been observed in the healthy brain and therefore has been proposed to have important physiological functions (228). However, the nitration of Y-18, Y-29, and Y-394 has only been observed in AD or other tauopathies (229). It is believed that nitration of these tyrosine residues alters tau conformation and reduces its ability to bind to microtubules (230). While there are some interesting insights on tau nitration, most of these studies are based on *in vitro* experiments. Therefore, studies focusing on this PTM will provide additional useful insights into its role in tau pathobiology.

## Effect of PTMs on Tau Degradation

Protein degradation is important for the maintenance of proteostasis and for preventing the accumulation of misfolded or aggregated protein species (231). There are two major protein degradation systems in the cell: the ubiquitin-proteasome system (UPS) and the autophagy-lysosomes pathway (231). Both protein degradation pathways are key effectors of the proteostasis network and are strongly influenced by PTMs. Protein clearance is highly regulated and utilizes ubiquitin as a tag for direct protein degradation in both proteasomal and autophagy systems. However, other PTMs also affect the process of protein degradation indirectly. This is the case for phosphorylation and acetylation, both of which can modify amino acid sequences known as degrons, which are important for protein degradation.

A subset of degrons are regulated by phosphorylation and thus are called phosphodegrons (232, 233). Phosphodegrons are short linear amino acid sequences, D/E-S/T-**G**XX-S/T-P or **LL**-S/T-**P**XX-S/T-P, where the phosphorylation of S/T residues promotes protein clearance through stimulation of subsequent ubiquitination and proteasomal degradation (232, 233). Proteins can also contain other motifs that promote degradation by the autophagy-lysosomes system. These pentapeptide motifs are known as KFERQ motifs as they are composed of one or two positively charged residues (K, R), one or two hydrophobic residues (I, L, V, F), one negatively charged residue (D, E), and one bookending glutamine (Q) (234). “Canonical” KFERQ motifs are complete without modification while “potential” KFERQ motifs require completion via a PTM such as phosphorylation (235) or acetylation (234, 236) (Figure 3).

Tau homeostasis is typically maintained via degradation by both UPS and autophagy-lysosome systems (96, 110). Yet at some point in the pathobiology of tauopathies, the local concentration of tau must increase to favor the formation of fibrils, oligomers, and aggregates. Does self-association of potentially abnormally modified tau lead to damage and subsequent impairment of protein degradation systems? In this model, tau accumulation is causal for neurodegenerative disease. Or, alternatively, does tau accumulation result from decreased efficiency or failure of the protein degradation systems? In this case, tau accumulation is a consequence of other pathobiology in neurodegeneration. This type of binary, chicken, or egg approach to the genesis of tauopathies may be over-simplifications of a complex, biological milieu that combines aspects of both tau accumulation as cause and consequence. Nonetheless, the answers to these questions are key, as they dictate certain nodes to target for disease therapy. In this section, we will delineate the pathways for tau clearance and describe how PTMs could play active roles in these processes.

### Tau Clearance by the Ubiquitin-Proteasome Pathway

The UPS pathway is responsible for the clearance of soluble, intracellular proteins (237, 238). The proteasome is a highly organized and complex molecular machinery responsible for the selective and efficient degradation of client proteins, whose basic science and function has been reviewed elsewhere (239–242). The 26S proteasome is responsible for the majority of protein clearance in mammalian cells and degrades proteins that are tagged with polyubiquitin chains (195). Therefore, the fact that tau is a target of ubiquitination (as discussed above) (109, 110) suggests that it can be degraded by the proteasome.

While ubiquitination is the proximal signal for protein degradation in the UPS, other PTMs such as phosphorylation are often required for tau ubiquitination to occur (243). For example, tau phosphorylated at proline-directed serine/threonine sites is selectively ubiquitinated by CHIP (103, 243), and thus phosphorylation in these sites increases tau ubiquitination and UPS-mediated degradation. Given that tau has six phosphodegron sequences (Figure 3), phosphorylation also regulates the ubiquitination and degradation of tau through this means (233). Phosphorylation of tau degrons is highly regulated by different kinases such as cdk5 and GSK3β (244), which work together in order to phosphorylate tau at specific residues and prepare the molecule for ubiquitination and degradation (245). However, phosphorylation of tau at alternative sites prevents ubiquitination and clearance. As a case in point, phosphorylation of tau at the KXGS motifs in the C-terminal microtubule binding repeat domains seems to prevent the ubiquitination and degradation of tau (243).

The dysfunction of the UPS and its related PTMs are associated with the development of tauopathies. Insoluble tau aggregates isolated from human AD brains contain proteasome subunits (246). Ubiquitin ligases such as CHIP and TRAF6 also colocalize with tau in AD brains (102, 103). Ubiquitinated tau is a component of NFTs (246). Further, a recent study from our group showed that the PSP-related *A152T* tau variant increases tau phosphorylation at threonine 153, potentially interfering with recognition of the local phosphodegron and thereby UPS degradation (233). Impairment of UPS activity has also been observed in postmortem human AD brains (246–250). While together these data implicate the UPS in tauopathies, they nonetheless represent correlative, rather than causal evidence. To our knowledge, no studies have established UPS failure as necessary and sufficient in promoting tau accumulation, aggregation, neuronal loss, and neurodegeneration.

### Tau Clearance by Autophagy

Autophagy is the process by which unnecessary or damaged cellular components are proteolytically degraded in the endosomal-lysosomal pathway. Based on the mechanisms by which the substrate is delivered for degradation, there are three major types of autophagy: chaperone-mediated autophagy (CMA), endosomal microautophagy (e-MI), and macroautophagy. Long-lived proteins or protein aggregates that are too large to be processed through the proteasome are typically degraded via autophagy (242). Tau, being a long-lived protein, is anticipated to be degraded in lysosomes (242) and indeed, it has been shown that both soluble and insoluble tau can be degraded by the three forms of autophagy (251, 252). Furthermore, it has been observed that the use of autophagy inhibitors, such as ammonium chloride (NH_4_Cl), chloroquine, and 3-methyladenine (3-MA), as well as the inhibition of lysosomal proteases, delays tau clearance, and enhances tau accumulation (253–255), confirming the importance of autophagy in tau degradation.

Not only is tau cleared by autophagy, but PTMs play distinct roles in modulating these processes. Within the amino acid sequence of the tau protein are found two KFERQ motifs (^336^QVEVK^340^ and ^347^KDRVQ^351^) (Figure 3). These consensus sequences direct tau toward either chaperone-mediated autophagy or endosomal-microautophagy (252, 256). In addition to the two canonical KFERQ motifs, tau contains four potential KFERQ motifs that can become canonical in the presence of phosphorylation, acetylation, or a combination of both (Figure 3) (*KFERQ finder)* (234), emphasizing that PTMs play an important role in the degradation of tau by these two systems. CMA-mediated protein degradation is a selective form of protein clearance in which the KFERQ motifs are recognized by the Hsc70 chaperone. Upon recognition, the proteins are transported to the lysosomal lumen after binding to the lysosome-associated membrane protein-2 (LAMP-2) (257). Tau protein can be efficiently degraded by CMA as long as the KFERQ motifs are not blocked by PTMs (252).

In addition to initiating CMA, the binding of Hsc70 to KFERQ motifs also targets cytosolic proteins for selective degradation by e-MI. This form of protein degradation occurs in late endosomes/multivesicular bodies instead of lysosomes and involves sequestering substrate proteins into intraluminal vesicles budding in toward the lumen of multivesicular endosomes (234, 256). Unlike CMA, in mammals, the presence of KFERQ motifs is necessary but not sufficient for e-MI (256). In addition to protein cargo delivery by Hsc70, the e-MI pathway relies on the ESCRT (endosomal sorting complexes required for transport) I and III complexes that are required for the formation of vesicles which internalizes the cytosolic cargo (256). A recent study has analyzed the influence of two different PTMs, oxidation and phosphorylation, on the e-MI-dependent degradation of tau (251). A substantial decrease in the association and internalization of tau in late endosomes via e-MI was observed upon oxidation of mutant tau, which suggests that the oxidation of tau at C291/C322 is a prerequisite for completing the internalization of tau through e-MI (251). Moreover, in the same study, the authors found that phosphorylating tau at S262, S293, S324, and S356 inside microtubule-binding domains diminishes its degradation by e-MI (251). In contrast, another study showed e-MI mediated degradation of total tau and of specific phosphorylated species (258). Thus, PTMs regulate the clearance of tau degradation by both CMA and e-MI.

Non-soluble, larger or aggregated species of tau are cleared via macroautophagy, a form of autophagy that involves the sequestration of cytoplasmic cargo into double-membrane vesicles known as autophagosomes, which fuse with the lysosome to degrade its contents (259). Several reports have confirmed that tau aggregates can be degraded by macroautophagy. For example, the aggregates generated by the overexpression of the tau repeat domain with an FTD-17 mutation (TauRDΔK280) (252) as well as the proteolytically cleaved tau isoform D421 (Tau-C3) (260) are degraded by macroautophagy in cell models of tauopathy. Interestingly, tau phosphorylated at KXGS motifs escapes degradation by the UPS but is efficiently degraded by macroautophagy (252), suggesting the that wild type tau can also be degraded by macroautophagy. As PTMs contribute to tau aggregation (see section Effect of PTMs on Tau Solubility and Aggregation), they likely regulate tau clearance via macroautophagy.

Failure of tau degradation by the autophagy-lysosomes pathway has been linked to tauopathies. For example, there are several studies suggesting a defect in macroautophagy in tauopathy patients (261–263). Furthermore, it has been shown that tau in NFTs colocalizes with lysosomes and lysosomal markers in human AD, CBD, and PSP brains (263–267), suggesting that tau is directed toward macroautophagy but fails to be degraded. Moreover, evidence from mouse and *Drosophila* models of FTLD-Tau indicates that the microtubule destabilization associated with the abnormal phosphorylation of tau leads to the disruption of the axonal vesicle transport by impairing the dynein-dynactin complex, vesicle trafficking, and autophagic flux (268, 269). Alterations in CMA may also contribute to tauopathies, since it has been described that tau carrying the FTD-related mutation P301L reduces tau's susceptibility for degradation by CMA (251). Overall, the regulation of the autophagy-lysosomes pathway seems to be essential for maintaining tau homeostasis in health and disease, and PTMs in tau influence the autophagic degradation of tau in multiple ways.

## Effect of PTMs on Tau Solubility and Aggregation

The degradation pathways discussed above represent a major component of the proteostasis network and ensure that, under normal conditions, tau is maintained in an optimally functional state (231). However, in tauopathies, there is a breakdown in tau homeostasis that in turn leads to aberrant accumulation, misfolding, and aggregation of tau (45, 242, 270). Recent studies suggest that PTMs contribute in sometimes surprising ways to altering tau solubility and thereby promoting its aggregation.

Various biophysical techniques have demonstrated that under physiological conditions, tau exists in a “natively unfolded” or “intrinsically disordered” state (45, 271, 272). Despite being intrinsically disordered, tau shows a preference for global interactions between its negatively charged N-terminus and positively charged repeat domains, leading to an energetically favored “paperclip” like conformation (273). In contrast to soluble tau, tau aggregates are composed of β-sheet rich structures known as amyloid fibrils (274–277). Amyloid fibrils are highly heterogeneous in nature and are not easily amenable to study via traditional methods used in structural biology. Recent breakthroughs in cryo-electron microscopy (cryo-EM) technology, however, have made it possible to determine high resolution three-dimensional structure of tau amyloid fibrils (278, 279). Determination of the atomic structures of tau aggregates may provide a better understanding of the mechanisms underlying their formation, spreading, and clearance.

To date, cryo-EM has elucidated the structure of tau filaments isolated from brains of patients with several different forms of tauopathies (279–284). According to these studies, each tauopathy is associated with a unique tau filament fold (279). The origin of such structural specificity is not yet known, but could emanate from PTMs (279). For example, tau is phosphorylated at S262 in tau filaments extracted from AD and CBD brains, but not in the filaments of PiD patients (11). Consistent with this phospho-modified residue contributing to aggregate structure, S262 is present in the tau filament core in PiD but not AD (279). Therefore, distinctive patterns of post-translational modification in different tauopathies may underlie not only the structural specificity of amyloids observed in cryo-EM structures, but could even contribute to selective vulnerability to these diseases.

To understand the fundamental role of PTMs in tau aggregation, it is important to account for the influence of PTMs over the charge composition and distribution within a polypeptide chain. The net charge on any protein is dependent on the content of ionizable groups. Net charge is 0 when the pH of the surrounding environment equals that protein's isoelectric point (pI). Similarly, net charge is positive at a pH below pI, and is negative at pH above its pI. Tau contains around 29 percent charged residues, has low hydrophobicity and has a low net charge of +2 at physiological pH. While tau is overall slightly positively charged, the amino-terminal residues (including the two N-terminal inserts) are more negatively charged, the microtubule-binding repeat region is predominantly positive, and the distal carboxy-terminal residues are mostly neutral (285). Such asymmetric distribution of charges has been proposed to play a crucial role in tau interactions with microtubules and other binding partners (45). It may also explain the inability of tau to undergo aggregation *in vitro* without polyanionic aggregation inducers (286–288). The inducers of tau aggregation are not typically present in neurons but their effect may be achieved through the masking of charged residues in tau via PTMs.

PTMs that make tau more negatively charged are phosphorylation, acetylation, and nitration. These PTMs, when present in the microtubule-binding domains, have been proposed to initiate tau aggregation by first weakening its interaction with negatively charged microtubules (26, 45, 289, 290) The reduced affinity of tau toward microtubules upon these modifications would increase the “free” pool of soluble modified tau molecules that might have a higher tendency to self-assemble into aggregates, as has been observed in case of phosphorylated tau (189, 291). The aggregation might be further promoted by electrostatic interactions between negatively charged, modified tau and positively charged, unmodified tau. The effects of phosphorylation on tau aggregation, however, seem to be site-dependent. For example, phosphorylation sites close to the N-terminus tend to prevent tau aggregation but phosphorylation sites in the vicinity of the C-terminus and microtubule-binding region seem to accelerate filament formation *in vitro* (285). A similar site-dependence has been observed in tau nitration, where depending on the residues that are modified, nitration can either promote or inhibit tau aggregation (230, 289). Interestingly, the core of tau filaments isolated from tauopathy patients contains multiple lysine residues that are susceptible to acetylation (280, 281, 283). Since the microtubule binding region constitutes a major part of the core of tau filaments, acetylation of lysine residues in this region likely plays an important role in the assembly of tau filaments.

Unlike phosphorylation, acetylation, and nitration that alter the charge of tau, methylation preserves the positive charge on lysine residues for a neutral effect on net charge of the protein. The role of methylation in tau aggregation is controversial and it is unclear whether tau methylation induces tau aggregation or has a protective role. While some studies have remarked that methylation at lysine residues facilitates abnormal protein aggregation (292, 293), further experimentation using recombinant tau has shown opposite results, demonstrating that methylation reduces the tendency of tau toward aggregation and promotes tubulin assembly (141). Interestingly, the methylation status of tau changes from dimethyl- to monomethyl-lysine with aging and disease (140). Therefore, it is possible that high stoichiometry (number of methyl groups/protein molecule) of methylation reduces the propensity of tau toward aggregation *in vitro* and promote tubulin assembly (140).

Another important modification in tau that does not affect charge but can affect tau aggregation is oxidation. The oxidation at one of the two cysteine residues C-322 (present in R3 repeat) (Figure 3) seems to promote tau aggregation into paired helical filaments (224). However, aggregation is inhibited under reduced conditions, upon mutation of cysteine to alanine and upon the formation of an intramolecular disulfide bond between C-322 and C-291 (224, 294).

While the PTMs described above involve the addition of small chemical groups to tau, other PTMs involve the addition of large sugar groups (glycosylation and glycation) and protein macromolecules (ubiquitination and SUMOylation) (Figure 2). These large molecules can have profound steric effects on the native conformation of tau and therefore can strongly influence misfolding and aggregation. The mechanisms by which glycation and glycosylation regulate tau solubility and aggregation are poorly understood. Glycation appears to promote the polymerization and stabilization of aggregated tau (295). Additionally, the N-glycosylation of tau is thought to be associated with maintaining the structure of NFTs (167). On the other hand, O-GlcNAcylation has been proposed to protect tau against aggregation (175). In fact, a study on tau peptides has shown that O-GlcNAcylation in proline-rich sequences favors a more disordered or extended conformation of tau (296).

Like sugars, the effects of ubiquitin and SUMO addition to tau on aggregation propensity have not been extensively studied. However, both mono- and polyubiquitination can contribute to the formation of insoluble tau inclusions (96, 117). In addition, a recent cryo-electron microscopy (cryo-EM) and mass spectrometry-based study performed on tau filaments isolated from the brains of tauopathy patients, has proposed that incorporation of ubiquitin into tau filaments mediates specific inter-protofilament packing by providing additional contacts between tau molecules in each protofilament via ubiquitin chains (282). SUMOylation is analogous to ubiquitination and has been shown to decrease the solubility of tau (128). However, the mechanisms by which SUMOylation reduces tau solubility and most likely promotes its aggregation, remain unknown.

Overall, PTMs on tau critically influence the assembly of natively unfolded tau into highly ordered β-sheet rich aggregates. It is likely that PTMs drive this process by altering the distinctive charge distribution on tau. It is worth emphasizing that while PTMs undoubtedly affect tau solubility and intermolecular interactions, the effect of PTMs on processes such as protein localization and degradation would also contribute, in a discrete and upstream manner, to when, how and why tau aggregation events occur.

## Cross-Talk and Competition Between PTMs

**Figure 4:**
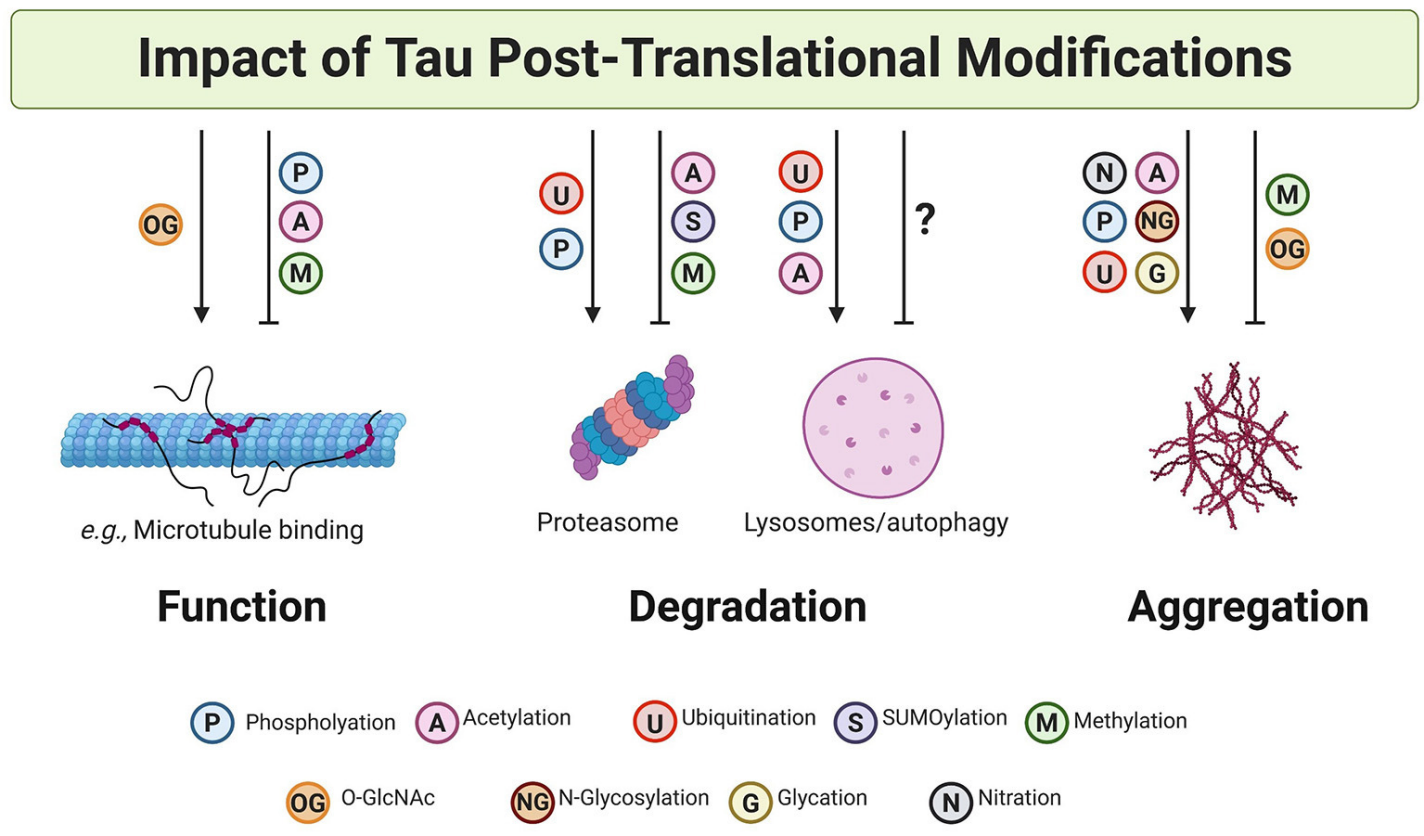
Post-translational modifications on tau impact tau function, degradation, and aggregation. PTMs can both promote and inhibit tau function, clearance, and aggregation. A non-exhaustive list of examples are illustrated here. In many cases, it is likely that cross-talk and competition between PTMs results in the specific and dynamic regulation of tau.

The PTM code hypothesis proposes that the combination of PTMs in proteins generates a dynamic “code” that can be translated into complex biological consequences (297). This hypothesis may apply to tau since it undergoes many modifications, often targeting the same amino acid residue (Figure 3). Such heterogeneity and competition between PTMs suggest that tau function and aggregation could be a consequence of the combination of several PTMs and/or the substitution of one PTM by another at the same position. In this regard, and based on the evidence described above, it is likely that while some PTM ensembles are essential for the regulation of tau function and degradation, others are more deterministic for formation of tau aggregates (Figure 4).

One level at which competing biological outcomes can be coded is via competition of different PTMs for the same amino acid. For example, S and T residues are modified by phosphorylation and O-GlcNAcylation. O-GlcNAcylation in some S/T residues protects tau from phosphorylation and thus blocks the effects of phosphorylation, which impacts microtubule-binding affinity and aggregation propensity, on these residues (155, 165, 298).

Perhaps even more interesting are lysine residues, which are susceptible to five different PTMs—acetylation, ubiquitination, methylation, SUMOylation, and glycation. Each one of these modifications have potentially different consequences for tau structure, function and aggregation. Since ubiquitination occurs on lysine residues, other lysine-based PTMs may prevent ubiquitination and therefore impair tau degradation (128, 139). In fact, many lysine residues that competent for ubiquitination are also targets for other PTMs (Figure 3). For example, K254 residue in tau can undergo ubiquitination and methylation. In fibrillar tau, K254 was found to be primarily methylated and ubiquitinated to a lesser extent, suggesting that methylation may block UPS-mediated tau degradation and lead to an increase in tau levels (89). Like methylation, acetylation could potentially prevent lysine ubiquitination, resulting in the insufficient turnover endogenous tau (72). The veritable log jam of PTMs that can be found on lysine residues marks this amino acid as a particularly interesting and likely important site at which cells orchestrate the biological outcomes they wish to achieve with tau and other proteins.

In addition to competition, crosstalk between PTMs can also be achieved through cooperativity. Some studies suggest that certain PTMs make tau more amenable to other modifications. *In vitro* tau SUMOylation seemingly enhances phosphorylation at residues such as T231 and S262 (128). Other studies have shown that tau methylation at K267 may result in subsequent and possibly abnormal phosphorylation of tau at S262 (89). It has also been proposed that N-glycosylation precedes tau phosphorylation (299). In each of these cases, though, a reasonable alternative interpretation is that SUMOylation, methylation, or N-glycosylation may impair tau clearance, enhancing the half-life of tau in the cytosol, and thus, providing more opportunities for phosphorylation.

On the other hand, phosphorylation can also affect the propensity of tau for other PTMs. For example, in specific experimental conditions the *in vitro* phosphorylation of tau in the microtubule binding domain activates tau autoacetyltransferase activity and therefore promote its acetylation (76). Furthermore, a protective role of the switch between tau phosphorylation and acetylation has been described. The acetylation of tau at KXGS motifs inhibits phosphorylation and thus prevents the detachment of tau from microtubules and its aggregation (34, 247). Additionally, the phosphorylation of tau at S422 seems to precede and block the cleavage of tau at D421 by caspase-3 (300), suggesting that phosphorylation at S422 could be a protective mechanism for the inhibition of the caspase-mediated cleavage of tau.

Taken together, the current literature suggests that crosstalk, combinations, and competition between PTMs play a critical role in tau function, degradation, and aggregation. An old truism states that “We are the company we keep.” If so, then future studies dedicated to documenting and decoding ensembles of tau PTMs will rise in importance in regards to understanding and developing interventions for tauopathies.

## Approaches and Limitations

The complete understanding of the role of PTMs in tau function and dysfunction is obstructed by several limitations in the methodology and models used. Historically, PTMs were identified using immunochemistry-based approaches. Using this method, several phosphorylation sites were identified and thus, a large number of anti-phospho tau antibodies are available (301). While immunochemistry methods are routinely used and are highly effective for the characterization tauopathies, they do not provide a comprehensive and quantitative description of PTMs, nor do they distinguish between single molecules with many PTMs vs. many molecules with single PTMs (302). Some of these problems can be solved by the use of quantitative mass spectrometry (MS) and nuclear magnetic resonance (NMR) spectroscopy, which provide a more detailed description of PTMs and allow the simultaneous analyses of a variety of PTMs (89, 110, 303–308). However, most of these methods are biased toward the peptides of highest intensity, and prevent the accurate determination of site-specific stoichiometry, associated site-specific dynamics and identification of labile PTMs like protonation (302). The high percentage of PTM sites in tau adds to the complexity, making it incredibly challenging to design and carry out such studies. To overcome some of these challenges, analytical methods including one known as FLEXITau (Full-Length Expressed stable Isotope-labeled tau) have been recently developed (309). This method allows for unbiased analyses of tau PTMs in a more quantitative manner. In this regard, a study using FLEXITau has reported 95 PTMs on tau isolated from postmortem human tissue from AD and has demonstrated that these modifications occur in an ordered manner and lead to tau aggregation (310).

The choice of models to use for studying PTMs also influences the understanding of the effect of PTMs in tau biology. The vast majority of PTMs have been identified *in vitro*, using biochemical assays where purified tau protein was incubated with specific enzymes (71) or using tau/enzymes overexpression systems in cell models (128). Some PTMs have also been confirmed or identified *in vivo* using tauopathy mouse models. However, most of these models are based on the expression of mutant forms human tau, and whether those findings and insights into PTMs extends to wild-type tau and sporadic tauopathies remains to be seen (79).

Tau PTMs have also been studied in post-mortem human brains using immunochemistry techniques and cryo-EM (282). It is important to keep in consideration that some PTMs can be lost or degraded during the process of brain extraction, fixation, storage, and analysis. Therefore, it is possible that in studies performed on human brain extracts, some tau PTMs may not be identified because of the caveats associated with brain preparation processes (37).

The cross-talk between PTMs is very important for the regulation of function, structure, and degradation of the proteins. Many studies on tau PTMs have focused only on one type of modification. Extrapolation of results obtained from single PTM studies lack context in regard to cross-talk and competition. As a result, a major caveat of many studies is the inability to differentiate between causes, consequences and bystander effects of these PTMs. The understanding of and solution to these limitations may enable the discovery of a “PTM code” in tau that will help to define sensitive biomarkers and lead to insights into disease pathobiology. In addition, unravelling the specific effects of PTMs in tau biology will pave the road toward new therapeutic approaches for tau-related neurodegenerative diseases.

## Concluding Remarks

PTMs are essential to the normal function of tau and therefore alterations in the pattern of PTMs has the potential to lead to tau dysfunction, accumulation, and abnormal aggregation. PTMs exert their function either through altering protein electrostatics or conferring steric alterations. Among all tau PTMs, phosphorylation has been studied most extensively and historically, was thought to hold primacy in regulating tau function and pathogenesis of tauopathies. However, increasing evidence suggests that many other PTMs contribute to dynamic regulation of tau. Cross-talk and competition between PTMs, in particular at lysine residues, introduce an intriguing new layer of complexity to orchestration of tau function and dysfunction. At the same time, combinatorial ensembles of PTMs may emerge as a means by which cells achieve differential biological outcomes involving tau. As a robust and fast-moving area of investigation, tau post-translational modification will likely impact the basic, pre-clinical, and clinical-translational aspects of tau biology in interesting ways for years to come.

## Author Contributions

CA and SA performed the literature review. CA, SA, and AK wrote the manuscript. All authors agreed to the final version of the manuscript.

## Conflict of Interest

The authors declare that the research was conducted in the absence of any commercial or financial relationships that could be construed as a potential conflict of interest.
